# GPS1 Exon 9 Mutations Represent a Rare Genetic Event in Penile Squamous Cell Carcinoma Pathogenesis

**DOI:** 10.3390/ijms27052460

**Published:** 2026-03-07

**Authors:** Lars Tögel, Felix Elsner, Olaf Wendler, Johannes Giedl, Nadine T. Gaisa, Georg Richter, Valentina Campean, Maximilian Burger, Bernd Wullich, Simone Bertz, Arndt Hartmann, Robert Stoehr

**Affiliations:** 1 Institute of Pathology, Universitätsklinikum Erlangen, Friedrich-Alexander-Universität Erlangen-Nürnberg (FAU), 91054 Erlangen, Germany; lars.toegel@uk-erlangen.de (L.T.); felix.elsner@uk-erlangen.de (F.E.); johannes.giedl@pathologie-weiden.de (J.G.); simone.bertz@uk-erlangen.de (S.B.); arndt.hartmann@uk-erlangen.de (A.H.); 2 Comprehensive Cancer Center Erlangen-EMN (CCC ER-EMN), 91054 Erlangen, Germany; olaf.wendler@uk-erlangen.de (O.W.); bernd.wullich@uk-erlangen.de (B.W.); 3 Comprehensive Cancer Center Alliance WERA (CCC WERA), 91054 Erlangen, Germany; 4 Bavarian Cancer Research Center (BZKF), 91054 Erlangen, Germany; 5 Zentrum Personalisierte Medizin Erlangen (ZPM-Erlangen), 91054 Erlangen, Germany; 6 Department of Otolaryngology, Head and Neck Surgery, Universitätsklinikum Erlangen, Friedrich-Alexander-Universität Erlangen-Nürnberg (FAU), 91054 Erlangen, Germany; 7 Institute of Pathology, University Hospital Ulm, 89081 Ulm, Germany; nadine.gaisa@uniklinik-ulm.de; 8 Institute of Pathology, RWTH Aachen University, 52062 Aachen, Germany; 9 Institute of Pathology, 31785 Hameln, Germany; richter@pathologie-richter.com; 10 Institute of Pathology, 91522 Ansbach, Germany; campean@patho-ansbach.de; 11 St. Josef Medical Centre, Department of Urology, University Regensburg, 93053 Regensburg, Germany; urologie@csj.de; 12 Department of Urology and Pediatric Urology, Universitätsklinikum Erlangen, Friedrich-Alexander-Universität Erlangen-Nürnberg (FAU), 91054 Erlangen, Germany

**Keywords:** penile squamous cell carcinoma, PSCC, molecular diagnostics, *GPS1*

## Abstract

Penile squamous cell carcinoma (PSCC) is rare, but a biologically aggressive malignancy. Recent comprehensive genomic profiling (CPG) efforts revealed the underlying genomic landscape of PSCC, identifying *TP53*, *TERT*, *CDKN2A*, *PIK3CA*, *NOTCH1*, and *FAT1* as frequently altered genes with potential roles in penile oncogenesis. In addition, recurrent mutations encoded in the *GPS1* gene have been observed in 7.4% of cases in a particular PSCC cohort. Functional studies demonstrated loss of function due to GPS1 Exon 9 missense mutations, proposing a possible role for these alterations as oncogenic driver events in PSCC. However, no other study confirmed the occurrence of *GPS1* gene mutations in PSCC. To elucidate the biological function of *GPS1* exon 9 mutations in PSCC pathogenesis, we utilized a comprehensive in-house cohort of 106 PSCC cases to explore their frequency and occurrence. Albeit, the previously reported *GPS1* mutations p.D382H and p.M384I were not observed in this large cohort of PSCC cases; this analysis, however, revealed two novel *GPS1* alterations in exon 9 in two (1.9%) of the analyzed cases: p.S372F (c.1115C>T) and p.A375D (c.1124C>A). This observation suggests that *GPS1* exon 9 sequence is a target of genetic alteration during PSCC pathogenesis. However, the non-recurrent nature of these alterations indicates that they are unlikely to represent oncogenic drivers in this disease.

## 1. Introduction

Penile cancer is a rare disease in industrialized countries, with an annual incidence of approximately 1 case per 100,000 men, but displays higher incidences in Southeast Asia, parts of Africa, and South America, with an estimated 37,700 new diagnoses worldwide in 2022 [1,2]. Penile squamous cell cancer (PSCC) accounts for around 95% of cancer cases, while melanoma, basal cell carcinoma, adenocarcinoma, urothelial carcinoma, and sarcoma make up the remaining histological subgroups [3]. PSCC is characterized by a multifactorial etiology, including HPV infection (contributing up to 50% of cases), smoking, lichen sclerosus, chronic inflammation, and poor hygiene [2,3,4]. When detected early, PSCC is curable by surgical resection in the vast majority of cases; however, PSCC is an aggressive disease, characterized by early metastasis to locoregional lymph nodes, which impacts prognosis and when distant metastases occur, 5-year survival rates decline to approximately 10–20% [2,3,5].

Comprehensive genomic profiling (CGP) employing next-generation sequencing (NGS) approaches, such as targeted sequencing or whole-exome/whole-genome sequencing (WES/WGS), enables extensive interrogation of cancer genomes to identify tumorigenic genetic aberrations, such as somatic mutations, copy number variants and structural rearrangements, as well as complex biomarkers, such as tumor mutational burden (TMB) and microsatellite instability (MSI). These insights have the potential to reveal tumor-specific driver mutations and define potential therapeutic targets, and may assist in tumor subtype classification, as well as unveil prognostic factors. While CGP has been utilized in common cancers, such as lung, colorectal, or pancreatic cancer, it has been only recently introduced into deciphering the molecular basis of penile cancer and, to date, still limited information on the underlaying molecular events driving PSCC oncogenesis is available. In a seminal study, McDaniel and coworkers employed targeted sequencing of 126 genes in a cohort of 43 PSCC cases and identified a median of two mutations per case, most frequently affecting *TP53*, *CDKN2A*, *PIK3CA*, and *HRAS*. Furthermore, they also observed recurrent gene amplifications of *MYC*, *CCND1*, *SOX2*, *ATP11B*, and *EGFR*, as well as *CDKN2A* gene locus deletions [6]. Subsequent targeted gene panel sequencing efforts comprising larger cohort sizes of up to 397 cases [7] and panels consisting of up to 592 cancer-related genes [8], along with the interrogation of all coding exons by WES [8,9,10,11], confirmed these initial findings, and, in addition, identified novel frequently mutated genes, including *TERT*, *FAT1*, *NOTCH1*, *FBXW7*, *CDKN2B*, *EP300*, *CASP8*, *NFE2L2*, *KRAS*, *KIT*, and *BRAF* [7,8,9,10,11,12,13,14,15]. These subsequent studies also revealed only a small fraction of PSCCs with high (10–19 mut/Mb), and very high TMB (≥20 mut/Mb) in approximately 9–10%, and 3–4.5% of cases, respectively [7,14]. Of note, HPV-related PSCC were more prevalent in TMB high/very high cases, and accumulated alterations in PIK3CA and KMT2D, whereas TP53 and CDKN2A associated predominantly with HPV-negative cases and low TMB [7,14]. Mismatch repair deficiency (dMMR) and MSI-high tumors were observed only in 0.5–1.1% of all cases [7,8].

In 2016, Feber and colleagues, applied WES analysis on 27 PSCC cases (23 pairs of PSSC matched to normal and 3 sole tumor samples) and revealed 810 mutated genes, of which 137 (17%) displayed recurrent alterations [10]. To identify putative driver genes commonly altered in PSCC oncogenesis, the authors screened for genes harboring high functional impact mutations that cluster within specific regions of the encoded protein. This analysis revealed *FAT1*, *TP53*, and the previously undescribed *GPS1* as candidate driver genes. The *GPS1* (G protein pathway suppressor 1) gene, also known as *COPS1*, *CSN1*, or *SGN1*, encodes for the protein COP9 signalosome complex subunit 1, which represents the largest and an essential component of the COP9 signalosome complex (CSN). The CSN is a conserved protease complex that interacts with hundreds of cullin-RING ubiquitin E3 ligases, decreasing their activity by removing the ubiquitin-like protein Nedd8 (deneddylation), from the cullin subunit [16]. The CSN is therefore an essential regulator of the ubiquitin (Ubl) conjugation pathway and is implicated in various cellular and developmental processes. In their cohort, Feber et al. observed two GPS1 mutated cases (7.4%), bearing mutations p.D382H, and p.M384I, respectively, both encoded in Exon 9 of the NM_212492 transcript isoform of GPS1. These mutations occur in the PCI (Proteasome, COP9, Initiation factor 3) domain, also called PINT motif, which serves as a scaffold for mediating and stabilizing protein–protein interactions [17]. Subsequent functional analysis revealed that GPS1 p.D382H and p.M384I overexpression disrupted miRNA-mediated gene silencing, and the authors concluded that GPS1 plays a tumor-suppressive role in PSCC oncogenesis [10].

To date, no other studies investigating the underlying molecular mechanisms of PSCC carcinogenesis have confirmed the presence of *GPS1* mutations in this disease, leaving the nature and functional relevance of *GPS1* alterations in PSCC unresolved. To define their occurrence and frequency, we performed Sanger sequencing on a cohort of archived PSCC cases. Consistent with earlier studies, our analysis did not detect the previously reported *GPS1* p.D382H and p.M384I variants but identified two novel alterations within exon 9.

## 2. Results

### 2.1. Cohort Characteristics

To delineate the landscape of *GPS1* (*CSN1*) mutations in penile squamous cell carcinoma (PSCC), we referred to an archival in-house cohort of 106 PSCC specimens collected at different sites throughout Germany. Some of these patient samples were previously used in a different study [18]. Table 1 summarizes the clinicopathologic characteristics of the current study cohort. The median patient age was 67.5 years (±11.7, Range 39–93 years, Table 1). Approximately 38% (40 of 104 analyzable tumors) of the cases were positive for HPV DNA using our two-step PCR approach.

**Figure 1:**
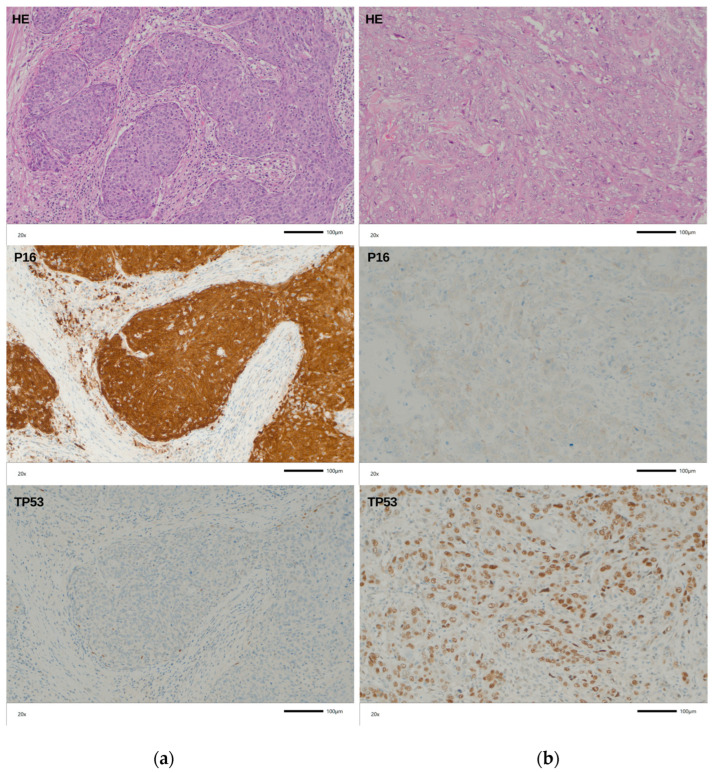
Hematoxylin and Eosin (H&E) staining (upper panel), as well as immunohistochemical staining of p16INK4a (middle panel) and TP53 (lower panel) of (**a**) a basaloid penile SCC tissue and (**b**) usual-type penile SCC tissue.

HPV subtyping showed the following distribution: HPV 18: n = 2; HPV16: n = 19; HPV 31: n = 1; HPV 33: n = 1; HPV 45: n = 1; HPV 31 + 52: n = 1; HPV 33 + 58: n = 1; no other specified: n = 14 (summarized in Table 2). The immunohistochemical analysis of p16INK4a expression (Figure 1a) showed a strong positivity in 37/80 (46%) cases. All 28 HPV PCR positive cases that were available on the TMA were also positive for a p16INK4a expression resulting in an overall HPV-positive status (positive PCR results and p16INK4a expression) of 37% in our cohort. The remaining 9/37 p16INK4a expression positive cases were negative for HPV DNA. Aberrant TP53 staining (Figure 1b) was found in 40/77 (52%) analyzable cases. In three cases, tissue spots of the TMA were lost during the staining process. Aberrant TP53 staining was observed in 28% (8/28) of HPV-positive cases and in 65% (32/49) HPV-negative cases. These results are in line with previously published data on the frequency of aberrant TP53 expression in PSCC [19] and the association of aberrant TP53 with HPV negativity [20].

### 2.2. Sanger Sequencing Analysis Enables High Detection Sensitivity of GPS1 Exon 9 Mutations

**Figure 2:**
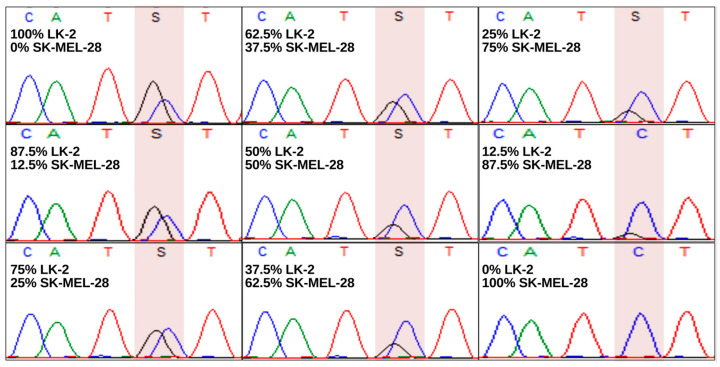
Sequential dilution of DNA from LK2 (GPS1 p.I366M) cells with DNA from SK-Mel28 cells (wild-type GPS1). GPS1 p.I366M mutation is detectable even in a background of >80% wild-type GPS1 DNA. The c.1098C>G nucleotide exchange is highlighted in red.

To our knowledge, *GPS1* (*CSN1*) is not included in any commercially available NGS gene panel. We therefore opted for Sanger Sequencing as it represents a reliable and cost-effective method suitable for the purpose of this study. Since we were unsuccessful in retrieving any control sample harboring the reported p.D382H or p.M384I mutation, we used DNA from the non-small-cell lung cancer (NSCLC) cell line LK-2 (Tebubio GmbH, Offenbach, Germany), carrying a p.I366M mutation (c.1098C>G) in exon 9 of *GPS1* (COSMIC sample ID: COSS687787) instead as positive control. DNA from the malignant melanoma cell line SK-MEL-28, which is a wild-type for *GPS1* (COSMIC sample ID: COSS905954), served as negative control. Following the verification of the *GPS1* exon 9 mutational status of LK2 and SK-MEL-28 cells, we next tested the sensitivity of our sequencing method by sequencing exon 9 of *GPS1* from a DNA mixture of the two cell lines across a serial dilution series. As shown in Figure 2, this analysis confirmed the high sensitivity of this sequencing approach, capable of detecting the *GPS1* c.1098C>G mutation in LK2 DNA within a mixture containing more than 80% wild-type SK-MEL-28 DNA.

### 2.3. Sequencing Analysis Reveals Two Novel GPS1 Exon 9 Mutations

**Figure 3:**
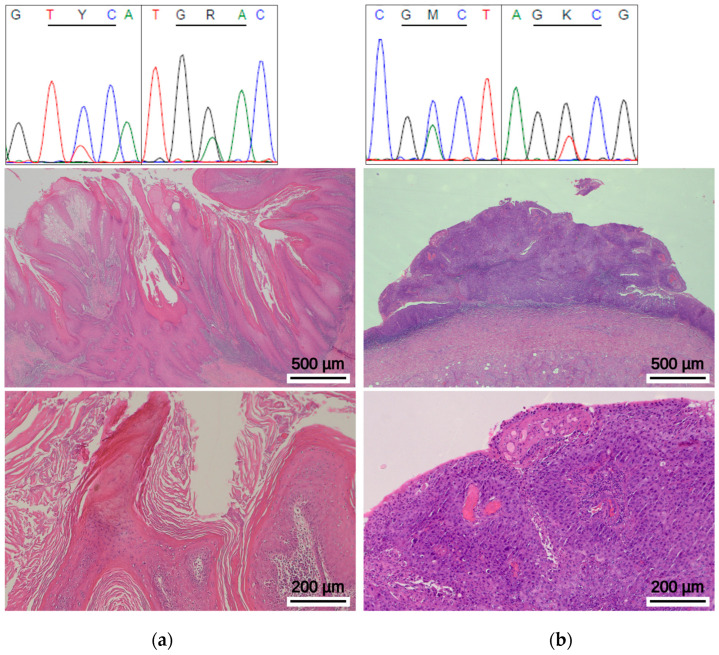
Novel *GPS1* exon 9 missense alterations identified in this study (**a**) upper panel: chromatogram of the *GPS1* p.S372F (c.1115C>T) alteration, left: sense direction, right: antisense direction. Middle and lower panel: representative H&E-stained tissue section of the verrucous PSCC case with the novel *GPS1* exon 9 p.S372F alteration (**b**) upper panel: chromatogram of the *GPS1* p.A375D (c.1124C>A) alteration, left: sense direction, right: antisense direction. Middle and lower panel: representative H&E-stained tissue section of the usual type (CIS) PSCC case with the p.A375D alteration.

Following the validation of the functionality and the sensitivity of the methodology, *GPS1* exon 9 sequencing was applied to the cohort. Sequencing of *GPS1* exon 9 was completed successfully for 104 of 106 cases (98%), while PCR failed for two samples due to poor DNA quality. None of the previously described p.D382H or p.M384I variants were detected in any of our cases. Instead, Sanger sequencing revealed previously unreported exon 9 alterations in two cases (n = 2/104, 1.9%). As shown in Figure 3a, a C to T substitution at nucleotide position 1115 (NM_212492.4:c.1115C>T), resulting in a serine(Ser, S)-to-phenylalanine (Phe, F) amino acid exchange at codon 372 (p.S372F), was identified in an HPV-negative verrucous PSCC (pT3G2, patient age: 49years). In addition, an HPV-negative usual-type PSCC (carcinoma in situ, CIS, patient age: 68 years) exhibited an A-to-C substitution at nucleotide position 1124 (NM_212492.4:c.1124C>A), resulting in an alanine (Ala, A) to aspartic acid (Asp, D) replacement at protein position 375 (p.A375D; Figure 3b).

**Figure 4:**
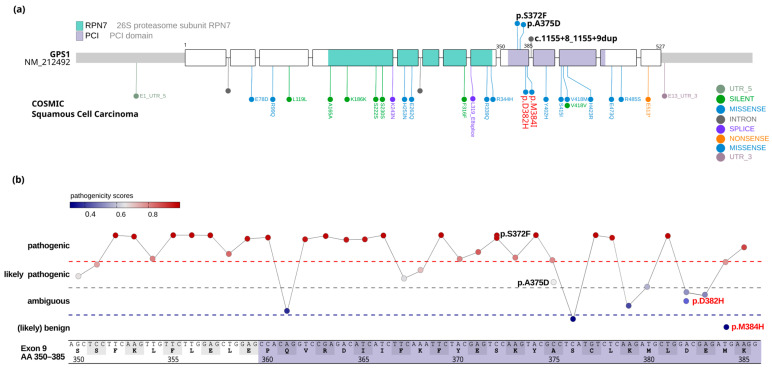
*GPS1* exon 9 gene mutations. (**a**) Schematic representation of the *GPS1* mRNA with coding exons depicted as boxes. Lollipops with amino acid changes shown in black indicate the localization of *GPS1* alterations identified in this study, amino acid alterations in red represent GPS1 variants identified by Feber et al. COSMIC database entries of known GPS1 alterations in squamous cell carcinomas are depicted by lollipops facing downwards from the exon/intron structure (visualized with ProteinPaint [25]). (**b**) Pathogenicity prediction scores of amino acids changes in all positions in exon 9. Displayed are the median AlphaMissense [26] pathogenicity prediction scores for all possible amino acid exchanges at positions 350 to 385 of exon 9 (data retrieved from https://doi.org/10.5281/zenodo.10813168). Pathogenicity scores for identified GPS1 alterations are depicted in conjunction with the corresponding amino acid alteration.

The p.S372F (c.1115C>T) and p.A375D (c.1124C>A) variants are previously undescribed amino acid substitutions within the PCI domain (amino acids 360–464; Figure 4a). This domain mediates the interaction of the GPS1 protein with other subunits of the COP9 signalosome complex [17], and, as suggested by binding assays, also appears to be essential for the interaction with various multi-protein complexes, including the SMC5/6 complex [21]. A query of the databases ClinVar [22], LOVD [23], and cBioPortal [24] yielded no entries for the identified genetic alterations, suggesting that GPS1 p.S372F and GPS1 p.A375D generally play no or only a minor role in tumorigenesis. The p.S372F variant is present with an allele frequency below 1% in the Genome Aggregation Database (gnomAD identifier: 17-80014227-C-T). Tools used for pathogenicity prediction yielded inconclusive results regarding the biological consequence of the p.A375D variant, whereas a potential deleterious effect was predicted for the p.S372F variant (Table 3 and Figure 4b). However, experimental studies are required to conclusively elucidate the impact of these genetic alterations on GPS1 functionality and, to date, the biological consequence and clinical relevance of these alterations are unknown.

### 2.4. Occurrence of GPS1 SNP rs34689427 in the PSCC Cohort Is Comparable to the SNP Frequency Observed in Population Studies

**Figure 5:**
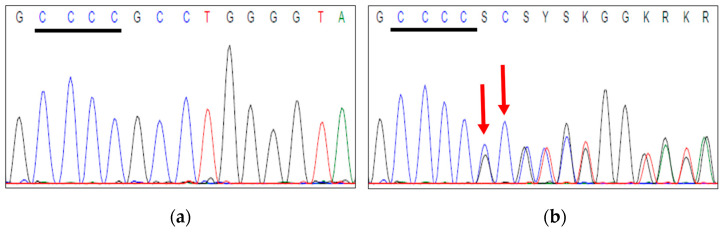
*GPS1* intron 9 polymorphism rs34689427. (**a**) PSCC case homozygous for the reference allele; (**b**) heterozygous PSCC case showing the reference allele and the alternative allele (c.1155+8_1155+9dup).

A previously reported single-nucleotide polymorphism (SNP) is localized in intron 9 of *GPS1* (rs34689427, c.1155+8_1155+9dup, Figure 3), which lies within the region covered by our Sanger sequencing approach (Figure 5). Interrogation of this sequence revealed 10 of 104 cases heterozygous for SNP rs34689427 representing an alternate allele frequency (AAF) of 9.6% in our cohort. This AAF is in line with the reported AAF of 10.1% for rs34689427 in the European cohort of the 1000 Genomes Project Phase 3 (https://www.internationalgenome.org/home), suggesting that the SNP re34689427 is not associated with an increased risk of developing PSCC.

## 3. Discussion

Comprehensive genome profiling has revealed the genomic landscape of penile squamous cell carcinoma (PSCC), identifying recurrently altered genes in penile tumorigenesis, including TP53, CDKN2A/B, PIK3CA, HRAS, TERT, EGFR, FAT1, NOTCH1, and FBXW7 [7,8,9,10,11,12,13,14,15]. Mutations in some of these genes may confer prognostic potential; for instance, alterations in TP53, CDKN2A, EGFR, and NOTCH1 have been associated with poor patient outcome, while PI3K pathway mutations were preferentially observed along improved progression-free survival [6,12,14]. In addition to these known cancer drivers, a single study described frequent inactivating mutations accumulating in Exon 9 of the GPS1 gene, suggesting that GPS1 exerts a tumor suppressor function in PSCC carcinogenesis [10].

The current study aimed to analyze GPS1 exon 9 alterations in a multi-institutional German cohort of 106 PSCC cases by targeted Sanger sequencing to conclusively elucidate the prevalence of GPS1 exon 9 genetic changes in this disease. While none of the previously reported GPS1 exon 9 variants (i.e., p.D382H and p.M384I) were observed in our analysis, we identified two novel GPS1 exon 9 alterations in this cohort instead. The observed mutation frequency of 1.9% in 104 successfully sequenced cases contrasts earlier findings by Feber et al., who observed a significantly higher prevalence (7.4% in 27 cases) in their test cohort. Several factors may explain this discrepancy, including sample size limitations of the prior test group, geographic or ethnic differences in patient populations, or methodological differences in sequencing approaches. In fact, low sequencing depth in WES analysis may contribute to the discovery of false positives, as suggested by Chahoud et al., who performed WES analysis on a cohort of 34 PSCC cases with a mean sequencing depth of 141× ([9] vs. 60× mean depth by Feber et al.), indicating that the employed methodology is critical in conclusively determining the mutational status of GPS1 exon 9 mutations. Nevertheless, the absence of recurrent GPS1exon 9 alterations in our study and the low overall mutation rate strongly suggest that GPS1 is not a commonly altered driver gene in PSCC carcinogenesis.

Our analysis revealed two novel GPS1 variants, i.e., p.S372F (encoded in c.1115C>T) and p.A375D (encoded in c.1124C>A), which map to the PCI (Proteasome–COP9–Initiation factor 3) domain of the protein. The PCI domain mediates protein–protein interactions and therefore represents an essential component in the functional composition of the COP9 signalosome (CSN) complex. In addition, the GPS1 PCI domain provides a binding interface to other multi-protein aggregates, such as the SMC5/6 complex, which plays a role in regulation of genome stability. This implies a biological rational for perturbations of GPS1 fostering PSCC carcinogenesis; nevertheless, due to the lack of observational data and inconclusive functional in silico predictions, the biological consequences of the p.S372F and p.A375D variants remain uncertain. The p.S372F variant has been observed in population-based datasets at very low allele frequency (<1%), while the p.A375D variant appears to be previously unreported. Taken together, both variants must currently be classified as variants of uncertain significance (VUS). Future studies are needed, such as knock-in cell lines or proteomic binding assays, to fully ascertain the functional consequences of the identified GPS1 exon 9 mutations.

Intriguingly, both novel GPS1 exon 9 alterations occurred in HPV-negative tumors with histomorphologies preferentially found in cases which arise through distinct molecular pathways compared to cases driven by HPV infection. However, given the rare occurrence and the small sample size, statistically meaningful conclusions cannot be drawn from our analysis and elucidating whether GPS1 exon 9 alterations characterize an HPV-negative PSCC subtype remains the subject of future studies employing larger cohorts with higher numbers of GPS1 exon 9 cases.

A methodological strength of our study is the high detection sensitivity of Sanger sequencing. Using the GPS1 c.1098C>G mutation LK2 cell line, we were able to demonstrate high detection capabilities even in cell mixtures containing more than 80% wild-type GPS1 DNA. This sensitivity underscores the robustness of the sequencing results and reduces the likelihood of missing low-frequency mutations.

Our sequencing methodology also allowed the determination of the prevalence of a known single-nucleotide polymorphism (SNP) in intron 9 of the GPS1 gene. This analysis revealed a frequency of 9.6% of SNP rs34689427 in the current PSCC cohort, which is consistent with the observed prevalence of 10.1% in the European population. This accidental finding clearly substantiates the near to real-world representativeness of our cohort and suggests that the presence of SNP rs34689427 confers no increase PSCC risk in healthy individuals.

This study has also limitations. To fully understand the biological significance of identified novel GPS1 exon 9 mutations, functional assays are mandatory, determining the pathogenicity of identified variants and discriminating them from genetic by-stander events in PSCC tumorigenesis. In this context, although our methodological approach has proven to be suitable and sufficient to fully analyze exon 9 alterations, it is necessary to include all GPS1 coding regions in future analyses in order to obtain a complete mutational profile of this gene in PSCC. Since GPS1 is part of a multi-protein complex, it is of interest whether GPS1 mutations are mutually exclusive with alterations in other components of the COP9 signalosome complex/COP9 signalosome pathway or whether GPS1 mutations coincide with other mutations in appreciated PSCC driver genes. Finally, patient acquisition for this study was limited to sites in Germany; therefore, geographical, and ethnical differences, as well as varying socioeconomic status, are not accounted for in this study.

Therefore, an increase in sample numbers collected at different regions and the utilization of comprehensive genomic profiling in combination with the utilization of functional assays is necessary to address these open questions in futures studies to conclusively elucidate the role of GPS1 mutations in PSCC.

Besides GPS1 mutation analysis we also examined the individual HPV status. This analysis revealed an overall HPV positivity (i.e., p16 expression and PCR positive) in 37% of our multicenter cohort samples. This result is in very good concordance with previously published data on HPV positivity in two other studies from Germany, which observed HPV infection in 38% and 30% of analyzed cases [5,27]. In contrast, pooled HPV DNA prevalence data from European and worldwide studies showed an HPV positivity frequency of approximately 50% in penile SCC [3,4]. In one line of this result, studies from Austria showed a very high HPV frequency ranging from 51 to 69% in two independent studies [28,29]. Meanwhile, a large Polish study revealed a relatively small HPV frequency of 14%, and likewise, a cohort study from France showed a linkage of penile SCC to HPV positivity in only 11% of the analyzed cases [30,31]. The different HPV positivity scores within this small compilation of European studies may be explained by the different methodological approaches applied by the authors. The low HPV frequency in the study from France might be explained by the fact that only morphological aspects of the tumors were considered to classify them as HPV-positive or -negative. The penile SCC cases from the Austrian studies were analyzed using the principle of reverse hybridization on strips carrying predefined specific oligonucleotide probes allowing the detection of a larger spectrum of high-risk HPV variants compared to our approach. Overall, the 2016 WHO classification of penile SCC based on HPV status could be reproduced very well within our cohort arguing for the robustness and validity of our data. Moreover, all of these data underline that there is a substantial HPV prevalence in Europe among men (approximately 22% [3,32]), especially in male genital cancer, which strongly endorses the introduction of HPV vaccination programs for boys and men. This fact was also confirmed by studies on the economical and public health outcome benefits of sex-neutral HPV vaccination [33,34].

Since p16 expression is frequently used as a surrogate marker for HPV infection, the HPV negativity in 24% (9 of 37) of p16-positive cases is indeed an interesting finding. However, this observation is not limited to our study cohort and actually represents a widespread molecular phenomenon. In their meta-analysis Olesen and coworkers revealed, by combing data of 13 independent studies (N = 999 cases), a pooled positivity of p16INK4a in 18.5% (range 9.6–29.6%) of HPV-negative cases [4]. The observed prevalence of p16INK4a-positive/HPV-negative cases of our study is within the range of published studies and is therefore a common molecular feature in some penile squamous cell carcinoma cases rather than a unique finding confined to our study.

Reasons for this discrepancy are manifold and may be explained by molecular events and/or technical issues. For instance, detection of HPV DNA can be affected by low viral copy number integration, exceeding the test’s detection limit, fragmentation of the viral DNA (especially in FFPE tissue), or by disrupting primer target regions due to unfavorable viral DNA integration (e.g., L1 gene loss). In addition, the CDKN2A gene expression may be upregulated/activated independently of HPV infection by transcriptional or epigenetic mechanisms or p16 posttranslational modifications upon oncogenic signaling [35,36,37]. While elucidating the molecular mechanisms of differential, case-specific p16 expression is indeed intriguing, it is not within the scope of the present work, and is therefore not extensively and conclusively discussed in the manuscript.

In summary, this study represents one of the first systematic efforts to assess GPS1 mutational status in PSCC and provides important insights into the prevalence of GPS1 exon 9 alterations in this rare disease. Our data suggests that GPS1 exon 9 mutations infrequently occur in PSCC, and the low mutation rate implies that they represent more likely genetic by-stander events than driving factors in PSCC carcinogenesis.

## 4. Materials and Methods

### 4.1. Patient Samples

The cohort was compiled from archival formalin-fixed, paraffin-embedded (FFPE) tissue samples obtained from 106 PSCC cases. Tumors were categorized and staged in accordance with the WHO classification of penile tumors [38] and the current AJCC/TNM-classification system [39]. As the current WHO classification of penile SCC incorporates the HPV status of the tumor (HPV-positive versus HPV-independent), all cases included in this study underwent analysis to determine the presence or absence of HPV, including subtyping. Detailed clinicopathological characteristics of the cases are presented in Table 1 and Table 2, and Figure 1.

### 4.2. Cancer Tissue Microdissection and DNA Extraction

Cancer tissue inspection, microdissection of tumorigenic tissue, and genomic DNA extraction was carried out as previously described [40]. In brief, tumor tissue was micro dissected from 5 µm formalin-fixed paraffin-embedded (FFPE) tissue sections, dewaxed, rehydrated, and briefly stained with 0.1% methylene blue. Tissue identity was confirmed against H&E-stained sections by a pathologist. Tumor cells (>80% purity) were collected under an inverted microscope, and genomic DNA was extracted using the Maxwell^®^ 16 Blood DNA Kit (Promega, Germany), following the manufacturer’s instructions.

### 4.3. PCR-Based Human Papillomavirus (HPV) Detection

HPV status of each PSCC sample was determined utilizing a two-step polymerase chain reaction (PCR) approach. First, HPV DNA was detected in general, without delineating subclasses using GP5+/6+ primers aligning to the HPV LI region [41]. In a second step, HPV subclasses were defined in positive cases by employing type-specific primers detecting HPV subclasses 11, 16, 18, 31, 33, 35, 39, 45, 52, 53, 58, 59, 66 and 68, as previously described [42,43].

### 4.4. Sanger Sequencing and Data Analysis

The sequence of *GPS1* Exon 9 was amplified by PCR in a total volume of 25 μL containing 150 ng DNA, 0.2 mM dNTP (Promega, Mannheim, Germany), 0.18 μM primers (sense: 5′-CAC TGG CCA CTT GGA GGG-3′; antisense: 5′-GGG GCC CAC CTC CAT CTC-3′, obtained from Metabion, Martinsried, Germany), and 0.0025 U/μL GoTaq (Promega). The thermal cycling conditions were as follows: initial denaturation for 3 min at 95 °C, 45 cycles of denaturation at 94 °C for 1 min, annealing at 62.4 °C for 1 min, elongation at 72 °C for 1 min and final primer extension at 72 °C for 10 min. Gradient PCR was used for the optimization of cycling conditions. After amplification, the resulting PCR product (size 195 bp) was purified using the Qiagen Dye Ex 2.0 TM Spin Kit (Venlo, The Netherlands) according to the manufacturer’s conditions. Sequence analysis was performed with PCR sense and antisense primers using a Big Dye Terminator v.1.1 Cycle Sequencing Kit and an ABI 3500 Genetic Analyzer (both Applied Biosystems, Foster City, CA, USA). Sequencing Analysis Software Version 5.4 from Applied Biosystems was used for sequencing chromatogram.

### 4.5. Tissue Microarry and Immunohistochemistry Analysis

A tumor tissue microarray (TMA) was constructed by extracting 1.2 mm tissue cores from available paraffin blocks of 80 cases, following published protocols to ensure standardized immunohistochemical analysis [44]. p16 and TP53 expression was assessed by immunohistochemistry on 5 µm sections using the BenchMark ULTRA autostaining system (Roche Diagnostics, Mannheim, Germany) with the iView DAB Detection Kit (Roche Diagnostics, Mannheim, Germany). Staining was performed with a monoclonal anti-p16 antibody (BD Pharmingen (Franklin Lakes, NJ, USA), clone G175-405, dilution 1:20) and a monoclonal anti-TP53 antibody (Dako (Glostrup, Denmark), clone DO-7, dilution 1:50). Slides were evaluated by a surgical pathologist (AH) blinded to clinical data. P16 expression was classified as positive when strong nuclear and cytoplasmic staining was present in >50% of cells [45]. TP53 positivity in the tumor was defined as strong nuclear staining in >10% of the cells or a complete absence of TP53 staining in the tumor cells (loss of expression [46]).

## Figures and Tables

**Table 1:** PSCC cohort clinicopathological characteristics and patient demographics.

	Cases (n = 106)
Age	years
Median age	67.5
Mean age	67.5 ± 11.7
Range	39–93
Tumor Stage	n
pTis	8
pT1a	42
pT1b	9
pT2	29
pT3	13
pT4	1
unknown	4
Tumor Grade	n
1	23
2	47
3	23
unknown	5
HPV Status (PCR)	n
positive	40
negative	64

**Table 2:** Histological subtypes of our PSCC cohort.

Histological Subtype	n (%)	HPV Neg. (PCR), n (%)	HPV Pos. (PCR), n (%)	Not Available, n
Carcinoma in situ	8 (7.5%)	-	6 (75%)	2
Non-HPV-related penile SCC				
Usual type	40 (38%)	35 (87.5%)	5 (12.5%)	
Verrucous	13 (12%)	13 (100%)	-	
Pseudohyperplastic	7 (6.6%)	6 (85.7%)	1 (14.3%)	
Carcinoma cuniculatum	1 (1%)	1 (100%)	-	
Sarcomatoid	1 (1%)	1 (100%)	-	
HPV-related penile SCC				
Basaloid	17 (16%)	3 (17.6%)	14 (82.4%)	
Warty-basaloid	10 (9.4%)	2 (20%)	8 (80%)	
Warty	3 (2.8%)	1 (33.3%)	2 (66.7%	
Clear cell	2 (1.9%)	1 (50%)	1 (50%)	
Lymphoepithelioma-like	2 (1.9%)	-	2 (100%)	
Unknown (n = 2; 1.9%)		1 (50%)	1 (50%)	

- = data not present.

**Table 3:** In silico variant effect prediction.

	NM_212492.4:c.1115C>T (p.S372F)	NM_212492.4:c.1124C>A (p.A375D)
AlphaMissense:	0.989 (likely pathogenic)	0.6608 (likely pathogenic)
REVEL:	0.517 (damaging)	0.302 (uncertain)
BayesDel:	0.214 (damaging)	0.235 (damaging)
Mistic:	0.35 (tolerated)	0.38 (tolerated)

## Data Availability

Dataset available on reasonable request from the corresponding author.

## Abbreviations

The following abbreviations are used in this manuscript:

PSCC	Penile squamous cell carcinoma
NGS	Next-generation sequencing
CGP	Comprehensive genomic profiling
WES	Whole-exome sequencing
WGS	Whole-genome sequencing
HPV	Human papilloma virus
CSN	COP9 signalosome complex

## References

[1] Filho A.M., Laversanne M., Ferlay J., Colombet M., Pineros M., Znaor A., Parkin D.M., Soerjomataram I., Bray F. (2025). The GLOBOCAN 2022 cancer estimates: Data sources, methods, and a snapshot of the cancer burden worldwide. Int. J. Cancer.

[2] Thomas A., Necchi A., Muneer A., Tobias-Machado M., Tran A.T.H., Van Rompuy A.S., Spiess P.E., Albersen M. (2021). Penile cancer. Nat. Rev. Dis. Primers.

[3] Brouwer O.R., Albersen M., Parnham A., Protzel C., Pettaway C.A., Ayres B., Antunes-Lopes T., Barreto L., Campi R., Crook J. (2023). European Association of Urology-American Society of Clinical Oncology Collaborative Guideline on Penile Cancer: 2023 Update. Eur. Urol..

[4] Olesen T.B., Sand F.L., Rasmussen C.L., Albieri V., Toft B.G., Norrild B., Munk C., Kjaer S.K. (2019). Prevalence of human papillomavirus DNA and p16(INK4a) in penile cancer and penile intraepithelial neoplasia: A systematic review and meta-analysis. Lancet Oncol..

[5] Mink J.N., Khalmurzaev O., Pryalukhin A., Geppert C.I., Lohse S., Bende K., Lobo J., Henrique R., Loertzer H., Steffens J. (2023). Evaluation of Prognostic Parameters to Identify Aggressive Penile Carcinomas. Cancers.

[6] McDaniel A.S., Hovelson D.H., Cani A.K., Liu C.J., Zhai Y., Zhang Y., Weizer A.Z., Mehra R., Feng F.Y., Alva A.S. (2015). Genomic Profiling of Penile Squamous Cell Carcinoma Reveals New Opportunities for Targeted Therapy. Cancer Res..

[7] Necchi A., Spiess P.E., Costa de Padua T., Li R., Grivas P., Huang R.S.P., Lin D.I., Danziger N., Ross J.S., Jacob J.M. (2023). Genomic Profiles and Clinical Outcomes of Penile Squamous Cell Carcinoma with Elevated Tumor Mutational Burden. JAMA Netw. Open.

[8] Nazha B., Zhuang T., Wu S., Brown J.T., Magee D., Carthon B.C., Kucuk O., Nabhan C., Barata P.C., Heath E.I. (2023). Comprehensive genomic profiling of penile squamous cell carcinoma and the impact of human papillomavirus status on immune-checkpoint inhibitor-related biomarkers. Cancer.

[9] Chahoud J., Gleber-Netto F.O., McCormick B.Z., Rao P., Lu X., Guo M., Morgan M.B., Chu R.A., Martinez-Ferrer M., Eterovic A.K. (2021). Whole-exome Sequencing in Penile Squamous Cell Carcinoma Uncovers Novel Prognostic Categorization and Drug Targets Similar to Head and Neck Squamous Cell Carcinoma. Clin. Cancer Res..

[10] Feber A., Worth D.C., Chakravarthy A., de Winter P., Shah K., Arya M., Saqib M., Nigam R., Malone P.R., Tan W.S. (2016). CSN1 Somatic Mutations in Penile Squamous Cell Carcinoma. Cancer Res..

[11] Wang Y., Wang K., Chen Y., Zhou J., Liang Y., Yang X., Li X., Cao Y., Wang D., Luo L. (2019). Mutational landscape of penile squamous cell carcinoma in a Chinese population. Int. J. Cancer.

[12] Monteiro F.S.M., Alencar Junior A.M., da Trindade K.M., Rebelatto T.F., Maluf F.C., Gazzola A.A., Barrios P.M., Bellmunt J., de Jesus R.G., Silva G.E.B. (2025). Molecular characterization of metastatic penile squamous cell carcinoma in developing countries and its impact on clinical outcomes: LACOG 2018 translational study. Oncologist.

[13] Jacob J.M., Ferry E.K., Gay L.M., Elvin J.A., Vergilio J.A., Ramkissoon S., Severson E., Necchi A., Killian J.K., Ali S.M. (2019). Comparative Genomic Profiling of Refractory and Metastatic Penile and Nonpenile Cutaneous Squamous Cell Carcinoma: Implications for Selection of Systemic Therapy. J. Urol..

[14] Hojny J., Hrudka J., Prouzova Z., Kendall Bartu M., Krkavcova E., Dvorak J., Michalkova R., Capka D., Zavillova N., Matej R. (2025). Altered TP53, CDKN2A, ATM, EPHA7, POT1, CHEK1, GRIN2A, and EGFR Predict Shorter Survival in Penile Squamous Cell Carcinoma. Mod. Pathol..

[15] Ferrandiz-Pulido C., Hernandez-Losa J., Masferrer E., Vivancos A., Somoza R., Mares R., Valverde C., Salvador C., Placer J., Morote J. (2015). Identification of somatic gene mutations in penile squamous cell carcinoma. Genes Chromosomes Cancer.

[16] Schulze-Niemand E., Naumann M. (2023). The COP9 signalosome: A versatile regulatory hub of Cullin-RING ligases. Trends Biochem. Sci..

[17] Tsuge T., Matsui M., Wei N. (2001). The subunit 1 of the COP9 signalosome suppresses gene expression through its N-terminal domain and incorporates into the complex through the PCI domain. J. Mol. Biol..

[18] Fiegl A., Wendler O., Giedl J., Gaisa N.T., Richter G., Campean V., Burger M., Simmer F., Nagtegaal I., Wullich B. (2024). Elevated Microsatellite Alterations at Selected Tetranucleotide Repeats (EMAST) in Penile Squamous Cell Carcinoma-No Evidence for a Role in Carcinogenesis. Curr. Oncol..

[19] Sand F.L., Lindquist S., Aalborg G.L., Kjaer S.K. (2025). The prognostic value of p53 and Ki-67 expression status in penile cancer: A systematic review and meta-analysis. Pathology.

[20] Tekin B., Whaley R.D., Collins K., Erickson L.A., Cheng L., Gupta S. (2026). Select updates in the pathology of kidney, testis, and penile cancer for 2026: Including FLCN-mutated (kidney) tumors, paratesticular mesothelial tumors, and TP53/HPV status in penile squamous cell carcinoma. Hum. Pathol..

[21] Horvath A., Rona G., Pagano M., Jordan P.W. (2020). Interaction between NSMCE4A and GPS1 links the SMC5/6 complex to the COP9 signalosome. BMC Mol. Cell Biol..

[22] Landrum M.J., Lee J.M., Riley G.R., Jang W., Rubinstein W.S., Church D.M., Maglott D.R. (2014). ClinVar: Public archive of relationships among sequence variation and human phenotype. Nucleic Acids Res..

[23] Fokkema I., Kroon M., Lopez Hernandez J.A., Asscheman D., Lugtenburg I., Hoogenboom J., den Dunnen J.T. (2021). The LOVD3 platform: Efficient genome-wide sharing of genetic variants. Eur. J. Hum. Genet..

[24] Cerami E., Gao J., Dogrusoz U., Gross B.E., Sumer S.O., Aksoy B.A., Jacobsen A., Byrne C.J., Heuer M.L., Larsson E. (2012). The cBio cancer genomics portal: An open platform for exploring multidimensional cancer genomics data. Cancer Discov..

[25] Zhou X., Edmonson M.N., Wilkinson M.R., Patel A., Wu G., Liu Y., Li Y., Zhang Z., Rusch M.C., Parker M. (2016). Exploring genomic alteration in pediatric cancer using ProteinPaint. Nat. Genet..

[26] Cheng J., Novati G., Pan J., Bycroft C., Zemgulyte A., Applebaum T., Pritzel A., Wong L.H., Zielinski M., Sargeant T. (2023). Accurate proteome-wide missense variant effect prediction with AlphaMissense. Science.

[27] Heller M., Prigge E.S., Kaczorowski A., von Knebel Doeberitz M., Hohenfellner M., Duensing S. (2018). APOBEC3A Expression in Penile Squamous Cell Carcinoma. Pathobiology.

[28] Ermakov M.S., Kashofer K., Regauer S. (2023). Different Mutational Landscapes in Human Papillomavirus-Induced and Human Papillomavirus-Independent Invasive Penile Squamous Cell Cancers. Mod. Pathol..

[29] Mannweiler S., Sygulla S., Beham-Schmid C., Razmara Y., Pummer K., Regauer S. (2011). Penile carcinogenesis in a low-incidence area: A clinicopathologic and molecular analysis of 115 invasive carcinomas with special emphasis on chronic inflammatory skin diseases. Am. J. Surg. Pathol..

[30] Czajkowski M., Falis M., Blaczkowska A., Rybarczyk A., Wierzbicki P.M., Gondek J., Matuszewski M., Hakenberg O.W. (2025). Penile Cancer Profile in a Central European Context: Clinical Characteristics, Prognosis, and Outcomes-Insights from a Polish Tertiary Medical Center. Cancers.

[31] Daubisse-Marliac L., Colonna M., Trétarre B., Defossez G., Molinié F., Jéhannin-Ligier K., Marrer E., Grosclaude P. (2017). Long-term trends in incidence and survival of penile cancer in France. Cancer Epidemiol..

[32] Coker K.L., Morgan E.L. (2026). High-Risk HPV in Men: A Hidden Threat to Public Health?. Rev. Med. Virol..

[33] Palmer C., Wahner C., Wolle R., Kreuter A., Klussmann J.P., Witte J., Luzak A., Reuschenbach M. (2025). Cost-effectiveness analysis of HPV vaccination of men who have sex with men in Germany. Infection.

[34] Qendri V., Bogaards J.A., Baussano I., Lazzarato F., Vanska S., Berkhof J. (2020). The cost-effectiveness profile of sex-neutral HPV immunisation in European tender-based settings: A model-based assessment. Lancet Public Health.

[35] Csergeova L., Krbusek D., Janostiak R. (2024). CIP/KIP and INK4 families as hostages of oncogenic signaling. Cell Div..

[36] Jiao Y., Feng Y., Wang X. (2018). Regulation of Tumor Suppressor Gene CDKN2A and Encoded p16-INK4a Protein by Covalent Modifications. Biochemistry.

[37] Li J., Poi M.J., Tsai M.D. (2011). Regulatory mechanisms of tumor suppressor P16(INK4A) and their relevance to cancer. Biochemistry.

[38] Moch H., Cubilla A.L., Humphrey P.A., Reuter V.E., Ulbright T.M. (2016). The 2016 WHO Classification of Tumours of the Urinary System and Male Genital Organs-Part A: Renal, Penile, and Testicular Tumours. Eur. Urol..

[39] Brierley J.D.G.M., O’Sullivan B., Rous B., Van Eycken E. (2025). TNM Classification of Malignant Tumours (UICC).

[40] Weyerer V., Schneckenpointner R., Filbeck T., Burger M., Hofstaedter F., Wild P.J., Fine S.W., Humphrey P.A., Dehner L.P., Amin M.B. (2017). Immunohistochemical and molecular characterizations in urothelial carcinoma of bladder in patients less than 45 years. J. Cancer.

[41] de Roda Husman A.M., Walboomers J.M., van den Brule A.J., Meijer C.J., Snijders P.J. (1995). The use of general primers GP5 and GP6 elongated at their 3′ ends with adjacent highly conserved sequences improves human papillomavirus detection by PCR. J. Gen. Virol..

[42] Lin C.Y., Chao A., Yang Y.C., Chou H.H., Ho C.M., Lin R.W., Chang T.C., Chiou J.Y., Chao F.Y., Wang K.L. (2008). Human papillomavirus typing with a polymerase chain reaction-based genotyping array compared with type-specific PCR. J. Clin. Virol..

[43] Sahiner F., Kubar A., Yapar M., Sener K., Dede M., Gumral R. (2014). Detection of major HPVs by a new multiplex real-time PCR assay using type-specific primers. J. Microbiol. Methods.

[44] Denzinger S., Burger M., Hammerschmied C.G., Wieland W.F., Hartmann A., Obermann E.C., Stoehr R. (2008). Pax-5 protein expression in bladder cancer: A preliminary study that shows no correlation to grade, stage or clinical outcome. Pathology.

[45] Riener M.O., Hoegel J., Iro H., Hartmann A., Agaimy A. (2017). IMP3 and p16 expression in squamous cell carcinoma of the head and neck: A comparative immunohistochemical analysis. Oncol. Lett..

[46] de Haan L.M., de Groen R.A.L., de Groot F.A., Noordenbos T., van Wezel T., van Eijk R., Ruano D., Diepstra A., Koens L., Nicolae-Cristea A. (2024). Real-world routine diagnostic molecular analysis for TP53 mutational status is recommended over p53 immunohistochemistry in B-cell lymphomas. Virchows Arch..

